# Predictive analysis across spatial scales links zoonotic malaria to deforestation

**DOI:** 10.1098/rspb.2018.2351

**Published:** 2019-01-16

**Authors:** Patrick M. Brock, Kimberly M. Fornace, Matthew J. Grigg, Nicholas M. Anstey, Timothy William, Jon Cox, Chris J. Drakeley, Heather M. Ferguson, Rowland R. Kao

**Affiliations:** 1 Institute of Biodiversity, Animal Health and Comparative Medicine, College of Medical, Veterinary and Life Sciences, University of Glasgow, Glasgow G61 1QH, UK; 2 London School of Hygiene and Tropical Medicine, Keppel Street, London WC1E 7HT, UK; 3 Global and Tropical Health Division, Menzies School of Health Research and Charles Darwin University, Darwin, Northern Territory 0810, Australia; 4 Gleneagles Kota Kinabalu Hospital, 88100, Kota Kinabalu, Sabah, Malaysia; 5 Infectious Diseases Society, Sabah-Menzies School of Health Research Clinical Research Unit, Kota Kinabalu 88560, Sabah, Malaysia; 6 Royal (Dick) School of Veterinary Studies and Roslin Institute, University of Edinburgh, Easter Bush Campus, Roslin, Midlothian EH25 9RG, UK

**Keywords:** disease ecology, zoonoses, malaria, *Plasmodium knowlesi*, boosted regression trees, disease occurrence prediction

## Abstract

The complex transmission ecologies of vector-borne and zoonotic diseases pose challenges to their control, especially in changing landscapes. Human incidence of zoonotic malaria (*Plasmodium knowlesi*) is associated with deforestation although mechanisms are unknown. Here, a novel application of a method for predicting disease occurrence that combines machine learning and statistics is used to identify the key spatial scales that define the relationship between zoonotic malaria cases and environmental change. Using data from satellite imagery, a case–control study, and a cross-sectional survey, predictive models of household-level occurrence of *P. knowlesi* were fitted with 16 variables summarized at 11 spatial scales simultaneously. The method identified a strong and well-defined peak of predictive influence of the proportion of cleared land within 1 km of households on *P. knowlesi* occurrence. Aspect (1 and 2 km), slope (0.5 km) and canopy regrowth (0.5 km) were important at small scales. By contrast, fragmentation of deforested areas influenced *P. knowlesi* occurrence probability most strongly at large scales (4 and 5 km). The identification of these spatial scales narrows the field of plausible mechanisms that connect land use change and *P. knowlesi*, allowing for the refinement of disease occurrence predictions and the design of spatially-targeted interventions.

## 1. Introduction

Infectious disease mapping plays a vital role in guiding public health policy and practice [1]. For diseases with environmental drivers, such as malaria, mapping has supported the ongoing and successful drive to reduce the number of infections worldwide and has been pivotal to understanding the effectiveness and progress of this effort [1–4]. As control reduces incidence, the geographical distribution of infection becomes more heterogeneous [5]. In situations where few data are available, predicted probability of disease occurrence can be mapped in place of measures such as incidence or prevalence. This approach has been applied to a variety of infectious disease systems using methods that combine the strengths of machine learning and statistics, originally developed to more accurately map species distributions in ecology (e.g. [6–8]). In addition to geostatistical mapping, disease occurrence mapping has helped describe the spatial distribution of infectious diseases worldwide, and provided information relevant to the design and execution of disease control programmes (e.g. [9–11]).

Ensemble boosted regression tree (BRT) analysis is one such method that is now widely used for disease occurrence mapping [6,11,12]. BRT analysis is increasingly used to identify patterns in large infectious disease datasets, building on analytical developments in macroecology [12–15], and has been used to generate hypotheses from these patterns [15]. BRT analysis combines decision trees, in which trees are grown with binary splits of predictor values to minimize prediction errors, and boosting, in which a collection of models are combined [16]. It allows for the uneven distribution of variation in predictor variables without the need for transformation, is not biased by correlation between predictors, can incorporate complex interactions and fit nonlinear functions [16].

A disadvantage of disease occurrence mapping is the difficulty identifying how different factors contribute to models that generate their spatial predictions; predictions may be sufficiently reliable, but it may not be clear why [14]. This is particularly problematic in relation to the scale of processes that could give rise to spatial heterogeneity of disease, as the environmental data used to predict occurrence are usually aggregated on a single spatial scale (e.g. square grid cells of 5 km × 5 km). This may be unavoidable if, for example, satellite data are only available at a fixed resolution, or census data are pre-aggregated over administrative units. However, even when disaggregated data are available at high resolution, there is often no evidence-based methodological recourse to guide decisions on the appropriate spatial scale for inclusion in models. Ecological processes occur at different spatial scales and the scale at which analyses of disease distributions are conducted influences the inferred contribution of the determinants of those distributions [17–19].

Differences between the spatial scales of the underlying biological processes that drive disease transmission and the scale imposed on models by the aggregation of predictor variables (such as into raster grid cells) is likely to be particularly influential in models of zoonoses and vector-borne diseases. Transmission dynamics of these diseases arise from the interaction of multiple species and the environment, probably occurring over a variety of spatial scales, which makes it less likely that predictors aggregated at a single spatial scale will capture important variation, especially if the influences of multiple scales are dependent on one another, and when few data are available [20].

*Plasmodium knowlesi* malaria is a vector-borne zoonosis in South East Asia, which usually infects long-tailed (*Macaca fascicularis*) and pig-tailed macaques (*Macaca nemestrina*) [21]. Transmitted by the *Anopheles leucosphyrus* group of mosquitoes, changes in forest cover impact vector habitats as well as macaque and human distributions [22]. Identified as a potentially lethal infection in humans and a major public health concern in 2004 [23], *P. knowlesi* is now the most common cause of malaria in Malaysia and parts of Indonesia, global hotspots of tropical deforestation [24–26]. It may be misdiagnosed or undiagnosed across South East Asia, and the World Health Organisation has advised it be incorporated into ongoing malaria elimination programmes [27]. Owing to this increasing public health concern, *P. knowlesi* was proposed as a global priority for disease mapping [4] and has since been mapped by BRT analysis, using historical data to highlight priority areas for surveillance [6].

This study introduces a novel approach to spatial scale analysis in disease occurrence prediction as a tool to identify the key scales that define the relationship between a zoonosis of serious public health concern (*P. knowlesi* malaria) and the rapidly changing landscape implicated in its spillover from macaques to humans in South East Asia. Where the highest numbers of cases have been reported (Malaysian Borneo), *P. knowlesi* incidence has been positively associated both with forest cover and historical forest loss [28]. However, the mechanisms of the proposed influence of deforestation on *P. knowlesi* transmission are unknown; for example, this could be owing to changes in macaque densities, vector bionomics or human behaviour. For the purposes of control, this precludes the assessment of which part(s) of the transmission cycle to target and which kind of interventions are most likely to be effective at which spatial scales. For example, if regulating land use change to reduce the proximity of macaque to humans, how far should regulated zones extend from planned or existing settlements? The spatial scales that define *P. knowlesi* occurrence identified by this study provide important hitherto missing information to inform such spatially targeted control measures.

## 2. Methods

### (a) Case and household data

Data on household locations of consenting polymerase chain reaction-confirmed *P. knowlesi* cases (*n* = 206) were obtained from a case–control study carried out between 2012 and 2014 in Kudat and Kota Marudu districts, Northern Sabah, Malaysian Borneo [29] and used as presence points. In this study, control households were selected in the vicinity of case households, making them unsuitable for use as absence points owing to spatial sampling bias. Instead, absence households were identified from the sampling frame of a cross-sectional survey geo-locating all households within 180 randomly selected villages in four districts in Northern Sabah (Fornace *et al.* [30]). Absence points were identified from households not reporting clinical *P. knowlesi* cases within the two districts included in the case–control study. These absence points were filtered so that there were no more than five per village, with the first absence point in each village sampled randomly, and the remainder chosen to maximize the total distance between absence points within that village to ensure spatial representativeness. Absence points were excluded if they were further than 5 km from a presence point (to prevent large areas being covered only by absences), nearer than 0.2 km to a presence point, or did not have permanent residents. Presence and absence points were excluded if they were located within an urban area, determined using administrative boundaries, as travel histories suggest cases reported in urban areas are unlikely to have been contracted in urban areas [29]. These filters resulted in a dataset including 206 presence points, 43 of which were located on the island of Banggi, and 1324 absence points, 105 of which were located on the island of Banggi. All household locations were visited and geolocated using a handheld global positioning system (GPS) (Garmin, USA).

### (b) Landscape variables

Data on forest cover at 30 m resolution were obtained from Hansen *et al.* [26], with annual forest cover defined categorically as over 50% canopy cover based on data derived from Landsat imagery. Although this definition of forest may not differentiate between forest and plantations, canopy cover has previously been associated with *P. knowlesi* incidence [28]. Cases were approximately evenly divided between 2013 (*n* = 101) and 2014 (*n* = 105), and as the annual classified satellite data composition method tracks back in time as far as necessary to find cloud-free imagery covering all locations, a frequent issue in Borneo [26], forest data was extracted from the 2014 annual composite as it was most likely to represent the environment contemporaneous with case reporting.

Scalable variables were extracted from forest cover data, including proportions of recent (previous year) and historical (previous 5 years) forest loss and cleared areas (table 1). Data on forest gain were only available aggregated over the period 2000–2012 and were included to represent types of land use distinct from straightforward forest persistence or clearance, such as agroforestry. Perimeter area ratio (P : A) was used as a proxy for fragmentation of these land cover categories, as variation in P : A was more evenly distributed across variables than other fragmentation measures.

**Table 1.** The 10 scalable landscape variables classified from Landsat satellite imagery used in the analysis [26]. (Grid cells estimated as greater than 50% tree crown cover density were defined as forested. Perimeter area ratio (P : A) was used as a proxy for fragmentation as variation in P : A was more evenly distributed across variables than any other measure.)

variable name	details	composite year
cover (previous year)	proportion of forested grid cells	2014
cover P : A (previous year)	perimeter area ratio of forested grid cells	2014
cleared (previous year)	proportion of non-forested grid cells	2014
cleared P : A (previous year)	perimeter area ratio of non-forested grid cells	2014
loss (previous year)	proportion of grid cells that changed from forested to non-forested	2014
loss P : A (previous year)	perimeter area ratio of grid cells that changed from forested to non-forested	2014
loss (previous 5 years)	proportion of grid cells that changed from forested to non-forested	2010–2014
loss P : A (previous 5 years)	perimeter area ratio of grid cells that changed from forested to non-forested	2010–2014
gain (all years)	proportion of grid cells that changed from non-forested to forested	2000–2012
gain P : A (all years)	perimeter area ratio of grid cells that changed from forested to non-forested	2000–2012
NDVI	normalized difference vegetation index, calculated from composite Landsat image	2014
NDVI SD	standard deviation of normalized difference vegetation index, calculated from composite Landsat image	2014
elevation	metres above sea level (ASTER global digital elevation model (GDEM))	2014
slope	maximum rate of change in elevation, calculated from ASTER GDEM	2014
population density	population density estimates	2010
aspect	direction of the steepest down slope (in degrees), calculated from ASTER GDEM	2014

Other environmental variables previously associated with malaria [31] were included as predictors in BRT models, including elevation, aspect and slope [32]. Average annual normalized difference vegetation index (NDVI), which quantifies the greenness of vegetation, was calculated from the Landsat imagery used as input for the Hansen *et al*. [26] 2014 classification. Additionally, the standard deviation of NDVI (SD NDVI) was also included, as variance in NDVI values in space may identify habitat type contrasts and boundaries. To address the possibility of reporting bias, the distance to the nearest clinic and the minimum distance to any road were included in a subset of BRT models. A list of clinics in the study area was obtained from the Ministry of Health, Malaysia, and all clinics and roads were geo-located using a hand-held GPS (Garmin 62s, Schaffhausen, Switzerland). All variables were extracted at 30 m resolution.

### (c) Spatial scales

Sixteen scalable variables (table 1) were summarized over buffer areas determined by a maximum overland distance of 0.1, 0.2, 0.5, 1, 2, 3, 4, 5, 7.5, 10 and 20 km (‘spatial scales’) from each household. Maximum overland distances (i.e. areas containing all grid cells less than the threshold overland distance from the focal household) were used rather than circular buffers to exclude parts of the landscape separated from focal households by water.

### (d) Ensemble boosted regression tree analysis

To balance the influence of presence and absence points [33] and quantify uncertainty [8], models were run on 100 datasets, each including all presence points (*n* = 206) and an equal number of randomly sampled (without replacement) absence points. To describe variation in the contribution of variables to predictive ability across scales, a model was fitted with all scalable variables included at all spatial scales (11 spatial scales and 16 variables giving 176 predictors). An additional model was fitted in which two non-scalable variables (shortest distance to clinic and road) were added (178 predictors). To compare overall predictive ability across scales, 11 ensemble models were fitted, one for each spatial scale (16 predictors each). A version of all models was fitted to data from the mainland only, excluding cases not on the main island of Borneo (e.g. on Banggi island) to examine whether these associations were impacted by the inclusion of households within smaller land areas.

Models were fitted by 10-fold cross-validation, dividing the dataset into 10 training sets with each comprising a unique combination of nine subsets of the data with the remaining subset withheld for independent validation [16]. Model predictive ability was assessed using area under the receiver operator curve (AUC). The tree complexity parameter of the boosted regression tree analysis was set at 5, so that each decision tree built as part of the model included five nodes, allowing for complex interactions between predictor variables. The learning rate, which determines the contribution of each decision tree to a BRT model, was tuned to between 0.0001 and 0.002 to minimize prediction error during cross-validation [23]. Marginal effect curves, the effect of the change in one unit of the predictor on the probability of disease occurrence, were plotted for all predictors by scale.

### (e) Relative variable importance

Profiles of relative variable importance (RVI) for landscape variables across spatial scales were derived from models that included all scales simultaneously so that the importance of scale variable-combinations could be assessed while accounting for the contributions of all other variable-scale combinations and interactions between them. RVI measures the number of times a variable is selected for splitting during the construction of a BRT model, weighted by the squared improvement of the model owing to the split, averaged over all trees in the model [16]. To aid the interpretation of RVI across scales within variables, Spearman rank correlation matrices comparing values between all pairwise combinations of scales were plotted for each variable.

To test whether peaks of RVI were driven by changes in variance available to BRT models across scales, variance was superimposed on RVI profiles. This is a necessary check, as if RVI tracked variance across correlated scales within variables, we could not preclude differences in RVI across scales arising owing to an artefact of available variance alone. To aid interpretation, variances were plotted as proportions of maximum variance across scales for each landscape variable. Relative variance was compared with median RVI using Spearman rank correlation tests across the whole study site.

### (f) Case clusters

To investigate whether analysis across spatial scales could be used to distinguish different sets of epidemiological circumstances driving *P. knowlesi* spillover, a cluster analysis was performed on the model fitted (whole-study-site, scalable variables only) marginal probabilities of occurrence for each scalable variable (*n* = 176) for all cases (*n* = 206). Cases were clustered into two groups using Ward's minimum variance method [34].

## 3. Results

### (a) Relative variable importance across scales

**Figure 1.**
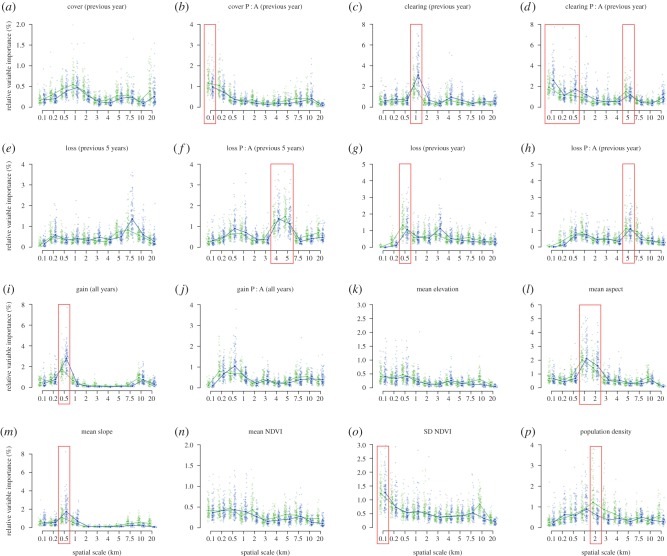
(*a*–*p*) Relative variable importance (RVI) of all variable-scale combinations from BRT models of *P. knowlesi* occurrence (176 predictors). See table 1 for variable definitions. Green points represent the whole-study-site, blue points the mainland-only model. Purple boxes indicate the 16 variable-scale combinations with the highest RVIs, detail of which is shown in the electronic supplementary material, figure S1a.

**Figure 2.**
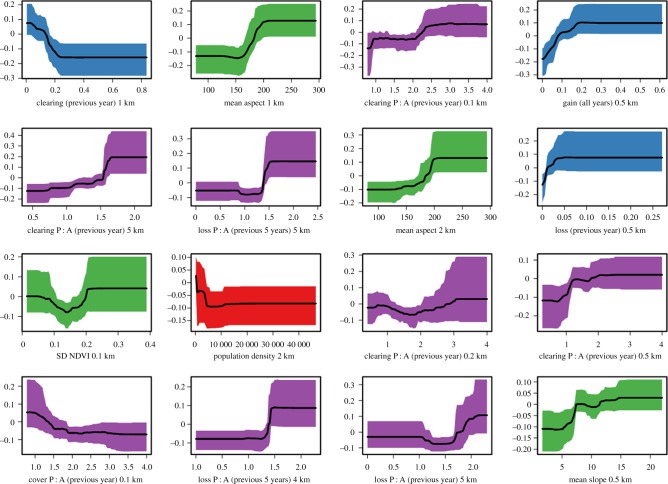
Marginal effect curves of the 16 variable-scale combinations with the highest relative variable importance across the whole study site (176 predictors).

RVI was extracted from an ensemble BRT model of *P. knowlesi* occurrence in Sabah, Malaysian Borneo, including 176 predictors and 16 scalable landscape variables (table 1) summarized at 11 spatial scales (figure 1). The emergent peaks in RVI profiles show that the influence of several variables on *P. knowlesi* occurrence prediction is strongly dependent on the spatial scale of their aggregation. The median relative importance of the proportion of cleared land was more than threefold higher when aggregated over a radius of 1 km from households than at any other scale in the mainland-only model, and more than twofold higher in the whole-study-site model (figure 1*c*). This was also the variable-scale combination with the highest RVI of the 176 predictors included in the whole-study-site model (electronic supplementary material, figure S1a). The corresponding marginal effect curve shows that probability of *P. knowlesi* occurrence was greater at lower proportions of cleared land within 1 km of households (figure 2).

The RVI profiles of five other variables included peaks at similar scales (figure 1 and table 1): mean aspect (1 and 2 km), mean slope (0.5 km), gain all years (0.5 km), population density (2 km) and loss previous year (0.5 km). The probability of *P. knowlesi* occurrence was predicted to be highest on west-facing slopes (higher aspect values, averaged over 1 and 2 km), which were relatively steep (averaged over 0.5 km), that both gained a relatively high proportion of canopy cover between 2000 and 2012 and lost a relatively high proportion of canopy in 2014 (both averaged over 0.5 km), and where (averaged over 2 km) few people lived (figure 2).

The fragmentation of forest loss was also an important predictor of *P. knowlesi* occurrence but only at relatively large spatial scales (e.g. 4–5 km, figure 1*f*,*h*). A similar pattern was observed both for the fragmentation of forest loss in the previous year (peak at 5 km) and in the previous 5 years (peaks at 4 km and 5 km), with the highest probability of *P. knowlesi* occurrence predicted when the landscape distribution of forest loss was most fragmented on these scales (figure 2).

The fragmentation of cleared land (as distinct from forest loss, see table 1) in the previous year was important at 5 km (figure 1*d*), as well as at three other scales (0.1, 0.2 and 0.5 km). The importance of three consecutive scales for one variable is likely to be owing to correlation across scales, and correlations were high in this case (electronic supplementary material, figure S3d). However, the correlation between small (0.1, 0.2 and 0.5 km) and large scale (5 km) aggregations was substantially lower (electronic supplementary material, figure S3d), which might suggest a real biological influence of this variable on two scales simultaneously. However, as the variance in this predictor variable was correlated with RVI (electronic supplementary material, figure S4) at small spatial scales, the possibility of their importance being artefactual at these scales cannot be ruled out, as higher variance is likely to lead to more frequent inclusion of variables in the decision trees that make up BRT models. The same interpretational caveat applies to the standard deviation of NDVI at 0.1 km (electronic supplementary material, figure S4).

### (b) Variance across scales

In general, the peaks of RVI (figure 1) do not arise from an artefact of correlation with variance (electronic supplementary material, figure S4 and table S1). However, in the case of the fragmentation of cleared land in the previous year, some caution is required in the interpretation of the importance of the smaller spatial scales. First, the comparison of variance with RVI across scales (electronic supplementary material, figure S4d) and their correlation (electronic supplementary material, table S1) suggest that RVI may be influenced by variance available to the model. Second, as the grid cells that make up the landscape variable layers are square, the perimeter length of patches will be overestimated at small scales [35]. In addition, the marginal effect curve for cleared P : A (previous year) at 5 km covers a greater range of predicted probability than those at the smaller scales of 0.1, 0.2 and 0.5 km (figure 2).

Although the standard deviation of NDVI at 0.1 km appears in the top 16 variable-scale combinations, the same caveat relating to changing variance across scales applies as above because RVI tracks variance (electronic supplementary material, figure S4). Therefore, it is possible that 0.1 km emerges as the most important scale owing to an artefact of variance available to the model, rather than owing to the influence of an underlying biological process on this scale. In addition, the marginal effect curve for SD NDVI 0.1 km does not suggest a strong influence on *P. knowlesi* occurrence probability (figure 2). The same applies to the importance of cover P : A at 0.1 km, as RVI tracks variance across scales (figure 2 and electronic supplementary material, table S1), and perimeters will be over-estimated at small scales.

### (c) Non-scaled variables

**Figure 3.**
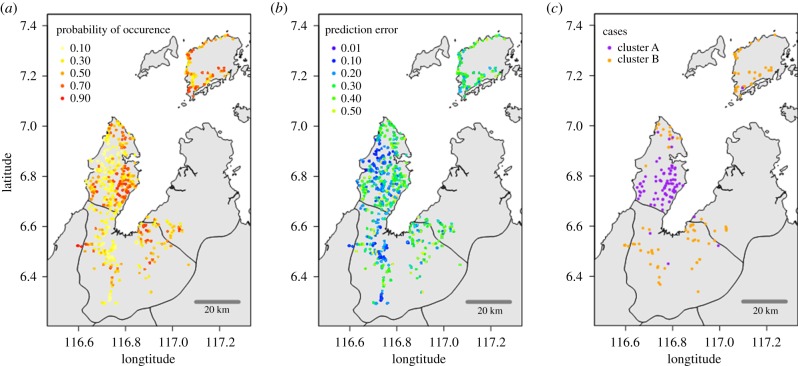
The locations of all households included in the study, showing (*a*) occurrence probability predictions from the whole-study-site model (176 predictors); (*b*) the prediction error from the same model; and (*c*) the location of the two clusters of case households.

The median prediction accuracy (AUC) of *P. knowlesi* occurrence across the whole study site was 0.76. The inclusion of two non-scalable variables, the shortest distance from households to the nearest clinic and road were included, increased this to 0.78. The shortest distance to road had the highest RVI in this model (electronic supplementary material, figure S1b), with the probability of *P. knowlesi* occurrence predicted to be highest at households furthest from roads (electronic supplementary material, figure S2). The addition of the two non-scalable variables only increased median AUC by 0.02, and gave rise to only minor changes in the most important variable-scale combinations (electronic supplementary material, figure S1) and negligible differences in their marginal effect curves (figure 2 and electronic supplementary material, figure S2). This suggests much of the variation explained by distance to roads and clinics is explained by included landscape factors; for example, distance to roads is probably highly correlated with population density and forest cover. This model was used to generate *P. knowlesi* human case occurrence predictions for all the households (figure 3*a*). The corresponding plot of prediction error by household shows there is little clustering of prediction error in space, and therefore that the model is not overly influenced by households in one area (figure 3*b*).

### (d) Case clusters

The division of case locations only (*n* = 206) by the marginal occurrence probabilities of the whole-study-site model into two clusters produced one cluster of 93 cases (cluster A) and another of 113 cases (cluster B). The two clusters appear to be spatially distinct, with cluster A mainly occurring on the mainland of the district of Kudat, and cluster B occurring on the island of Banggi and in the south of the Kudat peninsula (figure 3*c*). Exploration of the differences between clusters by examination of the 15 variable-scale combinations with the highest median marginal probability differences between clusters showed that cases in cluster A were characterized by low canopy cover, high proportion of cleared land and high population density at large spatial scales (electronic supplementary material, figure S5).

### (e) Prediction accuracy across scales

The ability of single-scale BRT models to predict *P. knowlesi* occurrence varied from an AUC of 0.55 (little better than a random model) to a maximum of 0.82. Models fitted to the smallest spatial scales had the lowest predictive power, those fitted to intermediate scales had the highest predictive power, and models that included all scales simultaneously performed better on average than all single-scale models (electronic supplementary material, figure S6).

## 4. Discussion

A key unanswered question about *P. knowlesi* transmission is what mechanism(s) give rise to the observed association between deforestation and human *P. knowlesi* incidence [28].

This study examines the influence of the absence of forest (cleared land), the process of forest loss, and the landscape distribution of forest loss (fragmentation) by spatial scale. This not only provides evidence that landscape fragmentation influences *P. knowlesi* spillover into humans, as it is thought to for other zoonoses such as Lyme disease [36] and Ebola [37], but also identifies the spatial scale of the influence of fragmentation on *P. knowlesi* transmission (within 4 and 5 km of households).

Consideration of the multiple spatial scales identified by this new analytical approach with corresponding marginal effect curves can suggest drivers of the observed patterns of disease occurrence. The effects of human, macaque and vector movement and density probably contribute to the spatial scale at which different landscape factors are predictive. For example, if individuals are exposed outside the house, the large-scale influence of the fragmentation of deforested areas (4–5 km) could emerge as a property of *P. knowlesi* spillover if humans commuted to fragmented deforested areas over distances of up to 5 km, and/or were at risk while there because of the nature of their work. This is consistent with the findings of a case–control study undertaken in the same area, including an increased risk of *P. knowlesi* (but not non-*P. knowlesi*) malaria in those walking to or from work or school [29]. Alternatively, macaque troops may respond to deforestation on this emergent scale, because they move distances of up to 5 km in response to fragmentation beyond a threshold, exposing households in sink areas to an increase in macaque density, which would be consistent with what estimates there are of *M. fascicularis* home ranges [38]. The step-like marginal effect curve of the fragmentation of deforestation on the probability of *P. knowlesi* occurrence suggests such a threshold effect. In addition, increasing values of the fragmentation of cleared land at 5 km predicted a similar step-like increase in occurrence probability. This suggests that the deforestation fragmentation result is not only an effect of the immediate disturbance of forest removal on *P. knowlesi* transmission, but one that is rather (or also) influenced by the habitat geometry it leaves behind [39]. Although 5 km was chosen as the maximum distance owing to village distribution and the small spatial scale of this study site (including islands), future work could explore whether landscape variables influence transmission at larger distances or explore the mechanisms behind these associations.

The probability of *P. knowlesi* occurrence was highest when the proportion of cleared land within 1 km of households was low. This suggests that households isolated in patches of forest or plantation (with less than 10% of the area within 1 km cleared) may be at the highest *P. knowlesi* exposure risk. This is in line with the traditional man-in-the-forest human *P. knowlesi* risk profile, which suggests that individuals who work on clearing forest or on plantations (usually adult men) are at highest risk of *P. knowlesi* infection, and additionally consistent with studies describing high vector densities in forest areas [22,40]. When averaged over this same scale, aspect also had an important influence on predicted *P. knowlesi* occurrence. Aspect is associated with *Plasmodium falciparum* infection in humans [31] but is identified here as a potential determinant of *P. knowlesi* human infection risk to our knowledge, for the first time. As households situated on west-facing slopes had the highest probabilities of disease, this may plausibly be because these households receive more sunlight in the afternoon, resulting in higher temperatures. For *P. falciparum*, increased temperature has been shown to shorten the duration of the incubation period in the mosquito or the length of the gonotrophic cycle, or speed up the development or increase the survival probability [41,42]. Alternatively, this association could arise through correlation between aspect and agricultural practice, with the peak of aspect RVI at 1 km arising from the way people modify (and the way both people and macaques use) agricultural land near households. *Plasmodium knowlesi* occurrence was also predicted to be higher at households on relatively steep slopes, which, as for aspect discussed above, could be a result of the influence of temperature on mosquito life history and infection dynamics, and/or the way that humans and macaques respond to slope. For example, if relatively steep slopes are uncultivatable, they may provide refuge from disturbance for macaques. That canopy regrowth (gain all years, table 1) had high RVI at the same scale as slope, suggests that peridomestic land use has an important influence over this scale, and therefore that the latter interpretation is more likely. Although this study has not equivocally identified mechanisms by which land use change influences human *P. knowlesi* infection risk, by mining the extra information contained within the spatial scale signatures of associations it has pared down the many plausible possibilities to a manageable number for further investigation. Future studies could additionally expand this analysis to evaluate the impact of different land use or forest types.

A challenge to a synthesis of *P. knowlesi* epidemiology across South East Asia is the considerable regional variation in infection patterns and risk profiles. The degree to which infection risk is concentrated in men who work in forests or plantations, the extent to which peridomestic transmission occurs, and whether human–vector–human transmission occurs under natural conditions are open questions [29,43,44]. Cluster analysis partitioned cases occurring in this part of Malaysian Borneo into two geographical groups, each with distinct risk profiles. Cluster A cases occurred at households around which there was relatively low forest cover, relatively high proportions of cleared land, relatively high population density, and that were immediately surrounded by fragmented forest cover compared with cluster B cases. These differences may reflect regional variation in the history of land use—the conversion of forest on the island of Banggi from the coast inwards, for example—and therefore the distinction between two sets of drivers of *P. knowlesi* spillover from macaques to humans. This novel approach to identifying transmission heterogeneities in disease occurrence datasets could be refined through integration with other sources of data, such as travel histories and human GPS tracking data, and developed into an effective tool for the surveillance of epidemiological transitions [45].

## 5. Conclusion

The consideration of multiple spatial scales can add value to analysis of disease occurrence by delivering more accurate spatial predictions, and identifying the key spatial scales of transmission. In the case of *P. knowlesi*, the application of a data mining approach has teased apart the potentially conflicting influences of forest cover and forest loss [28] on disease occurrence, identifying the latter as an effect of fragmentation on relatively large spatial scales and the former as an effect of the proportion of cleared land nearer to households. This could provide the key to the prediction of disease risk under models of future land use, and the design of spatially-targeted disease interventions. This new scale-focused approach could be widely applied to other zoonoses and vector-borne diseases of public health concern.

## Supplementary Material

Supplementary information

## Data accessibility

All analyses were performed in R, and code and sample environmental data are available at: https://github.com/kfornace/monkeybar. Owing to data confidentiality, human disease and household data are available through contacting relevant ethics committees as described in [29,46].
