# Supplementary material for: Predictive analysis across spatial scales links zoonotic malaria to deforestation
Source: Proc Biol Sci. 2019 Jan 16;286(1894):20182351. doi: 10.1098/rspb.2018.2351 (PMC6367187; doi:10.1098/rspb.2018.2351)
Supplement: Supplementary information [file rspb20182351supp1.pdf]

SUPPLEMENTARY MATERIAL

Predictive analysis across spatial scales links zoonotic malaria to deforestation

Patrick M. Brock, Kimberly M. Fornace, Matthew J. Grigg, Nick M. Anstey, Timothy William, Jon Cox, Chris J. Drakeley, Heather M. Ferguson, Rowland R. Kao

Supplementary Material Content

Figure S1: Top 16 predictors

Figure S2: Marginal effect curves of full model including non-scalable variables

Figure S3: Correlations across scales

Figure S4: Variance with relative variable importance

Figure S5: Case cluster profiles

Figure S6: Single-scale model predictive ability by scale

Table S1: Correlations of variance with relative variable importance

Figure S1. Relative variable importance (RVI) of the 16 most important variable-scale combinations from a) the full BRT model fitted across the whole study site (176 predictors); and b) the full BRT model fitted across the whole study site including two additional non-scalable variables – shortest distance to road and clinic – (178 predictors).

a) b)

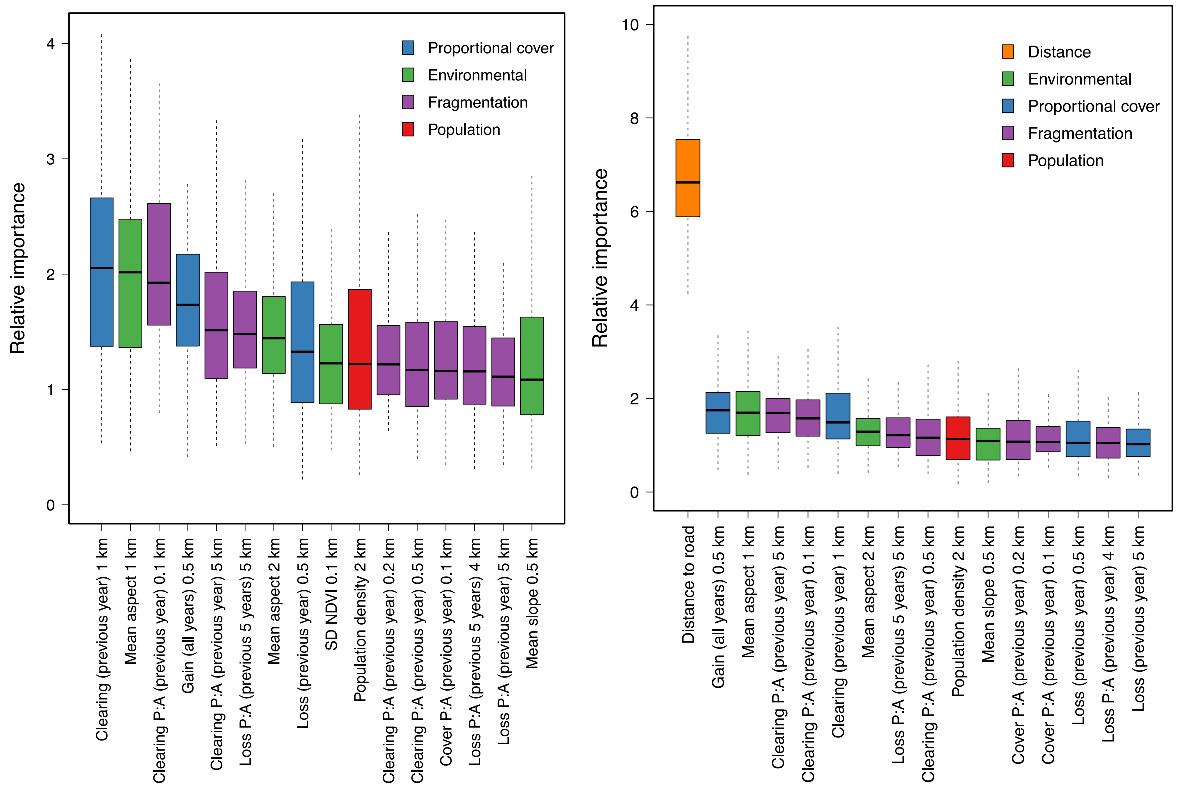

Figure S2. Marginal effect curves of the 16 variable-scale combinations with the highest relative variable importance (RVI) fromthe full BRT model fitted across the whole study site including two additional non-scalable variables – shortest distance to road and clinic – (178 predictors).

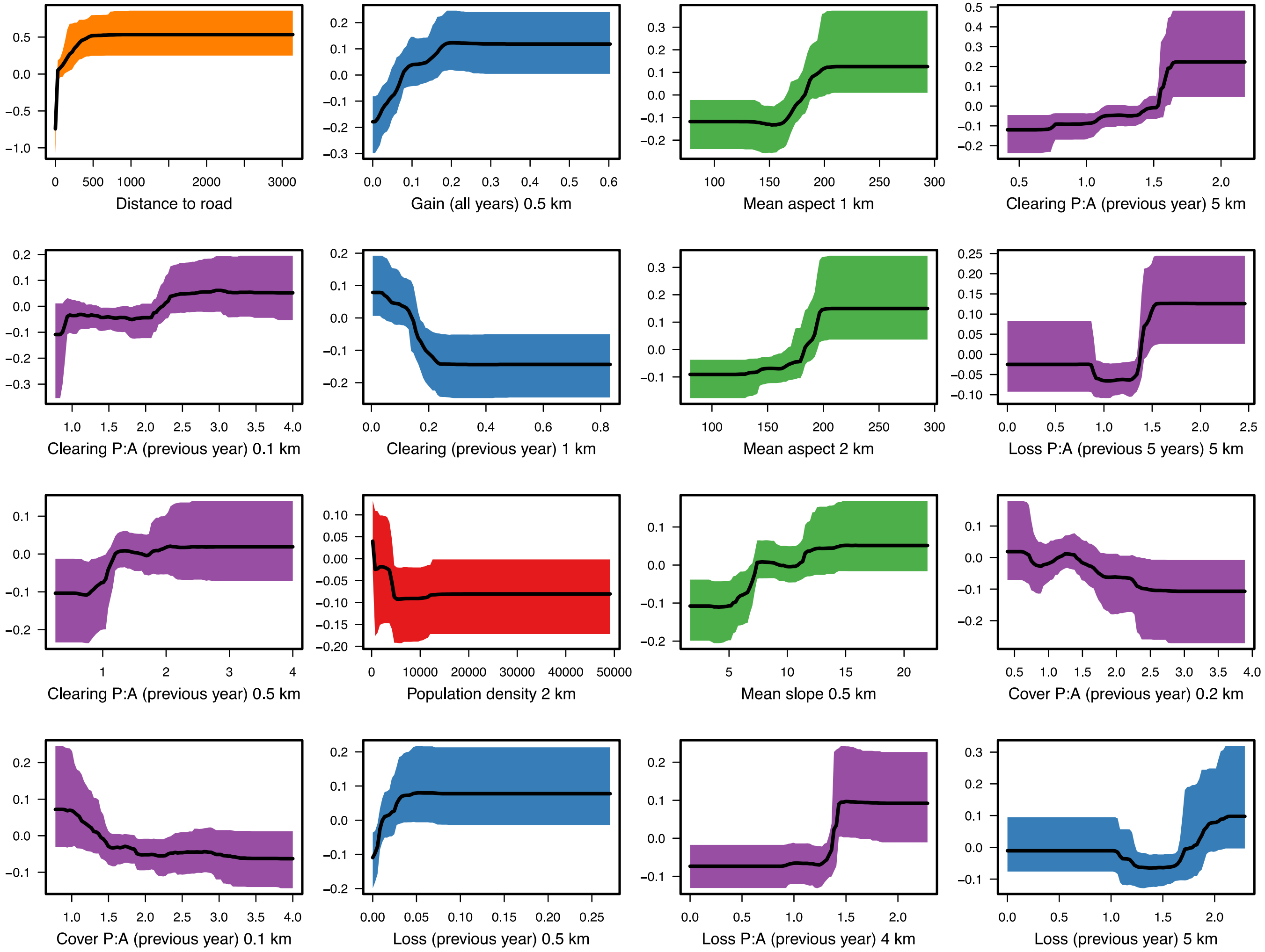
Distance to road
Gain (all years) 0.5 km
Mean aspect 1 km
Clearing P:A (previous year) 5 km
Clearing P:A (previous year) 0.1 km
Clearing (previous year) 1 km
Mean aspect 2 km
Loss P:A (previous 5 years) 5 km
Clearing P:A (previous year) 0.5 km
Population density 2 km
Mean slope 0.5 km
Cover P:A (previous year) 0.2 km
Cover P:A (previous year) 0.1 km
Loss (previous year) 0.5 km
Loss P:A (previous year) 4 km
Loss (previous year) 5 km

Figure S3. Spearman rank correlations between scales for all 16 scalable landscape variables.

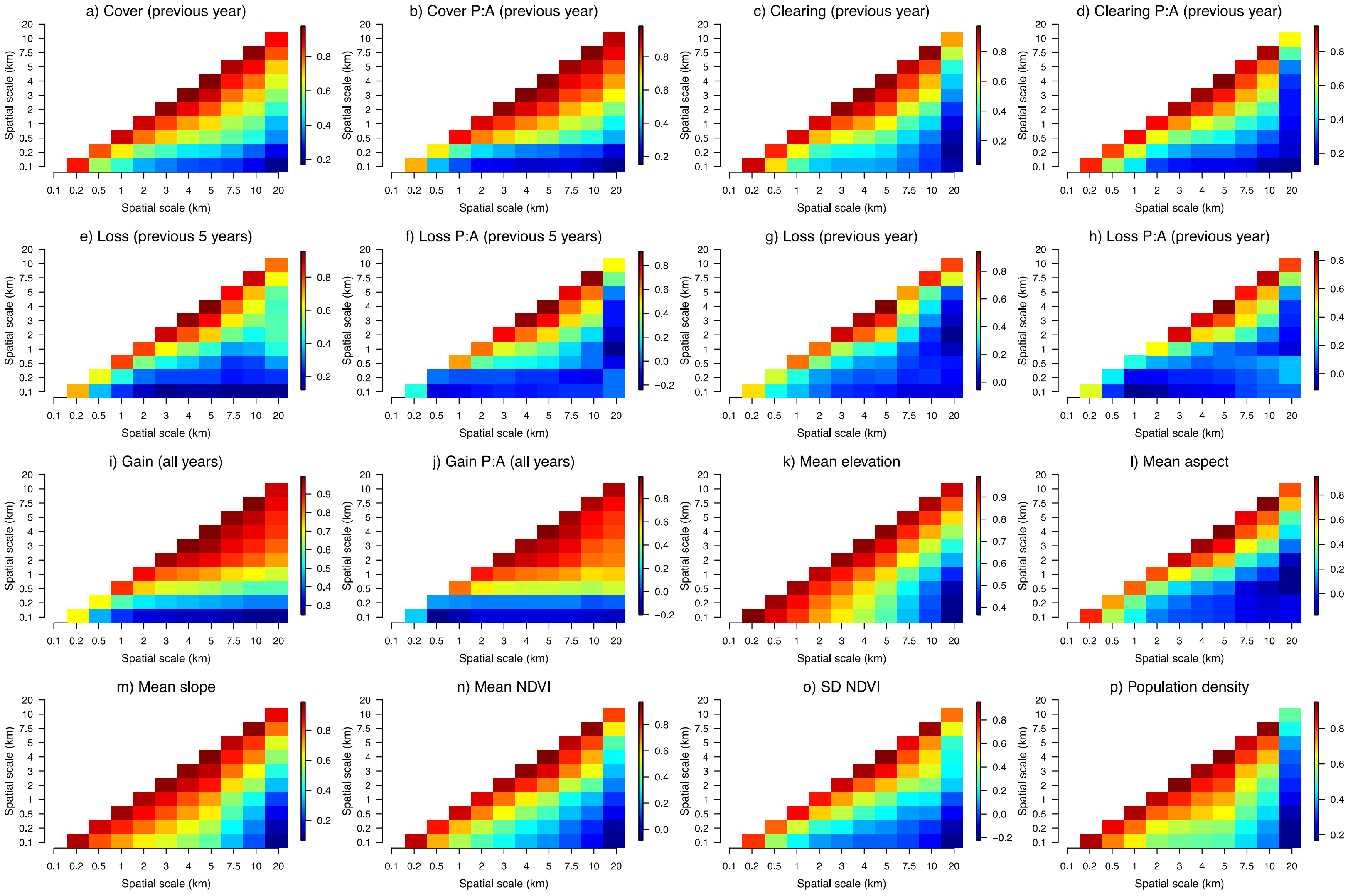

Figure S4. Relative variances superimposed on the traces of relative variable importance (RVI) of all variable-scale combinations (as shown in Figure 2) from full BRT models of *P. knowlesi* occurrence (176 predictors). Green points represent the whole study site, blue points mainland-only data.

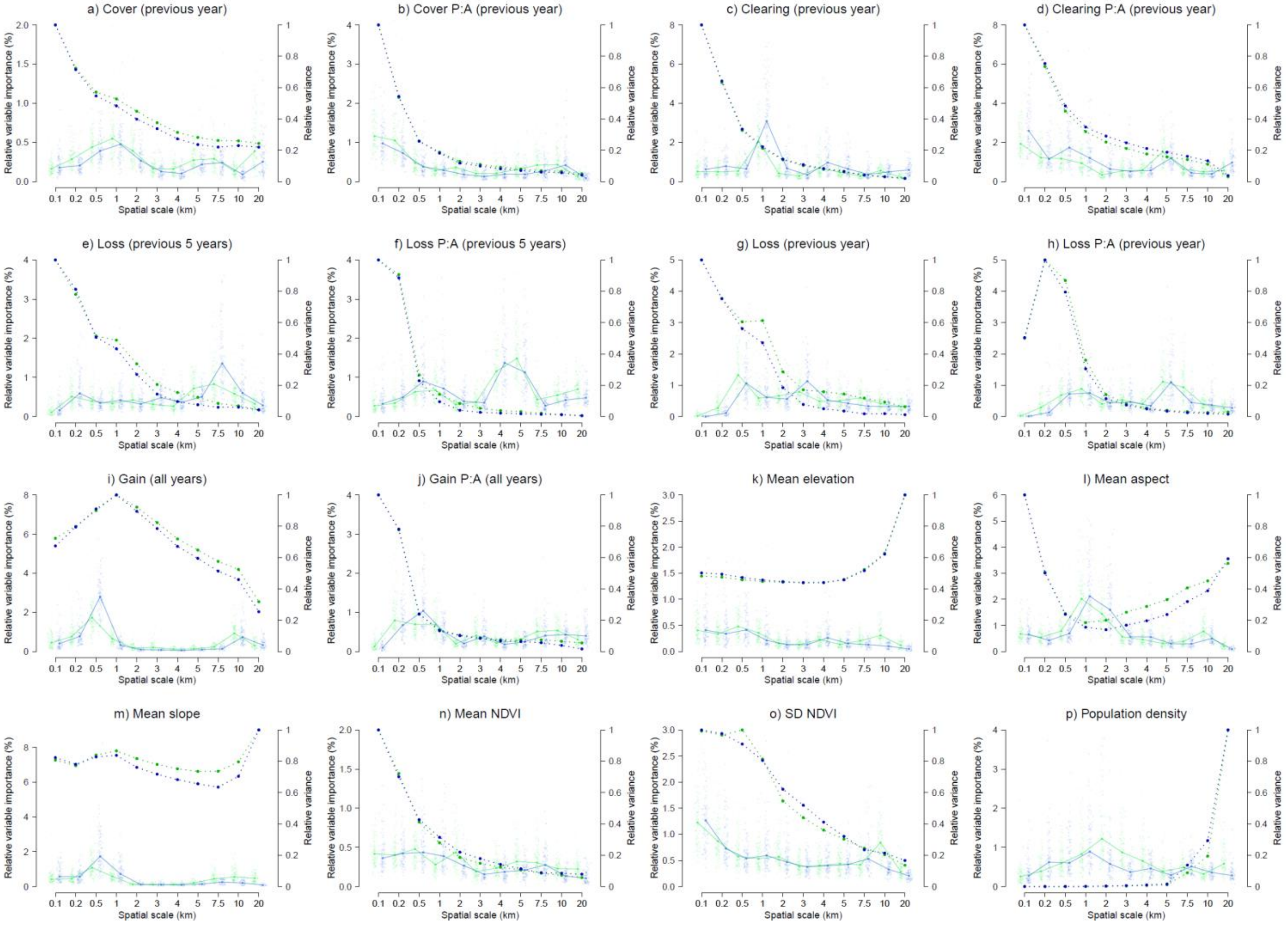

Figure S5. Variation in the 15 variable-scale combinations with the greatest differences in median marginal probability of occurrence between clusters. Cluster A shown in purple, cluster B shown in orange.

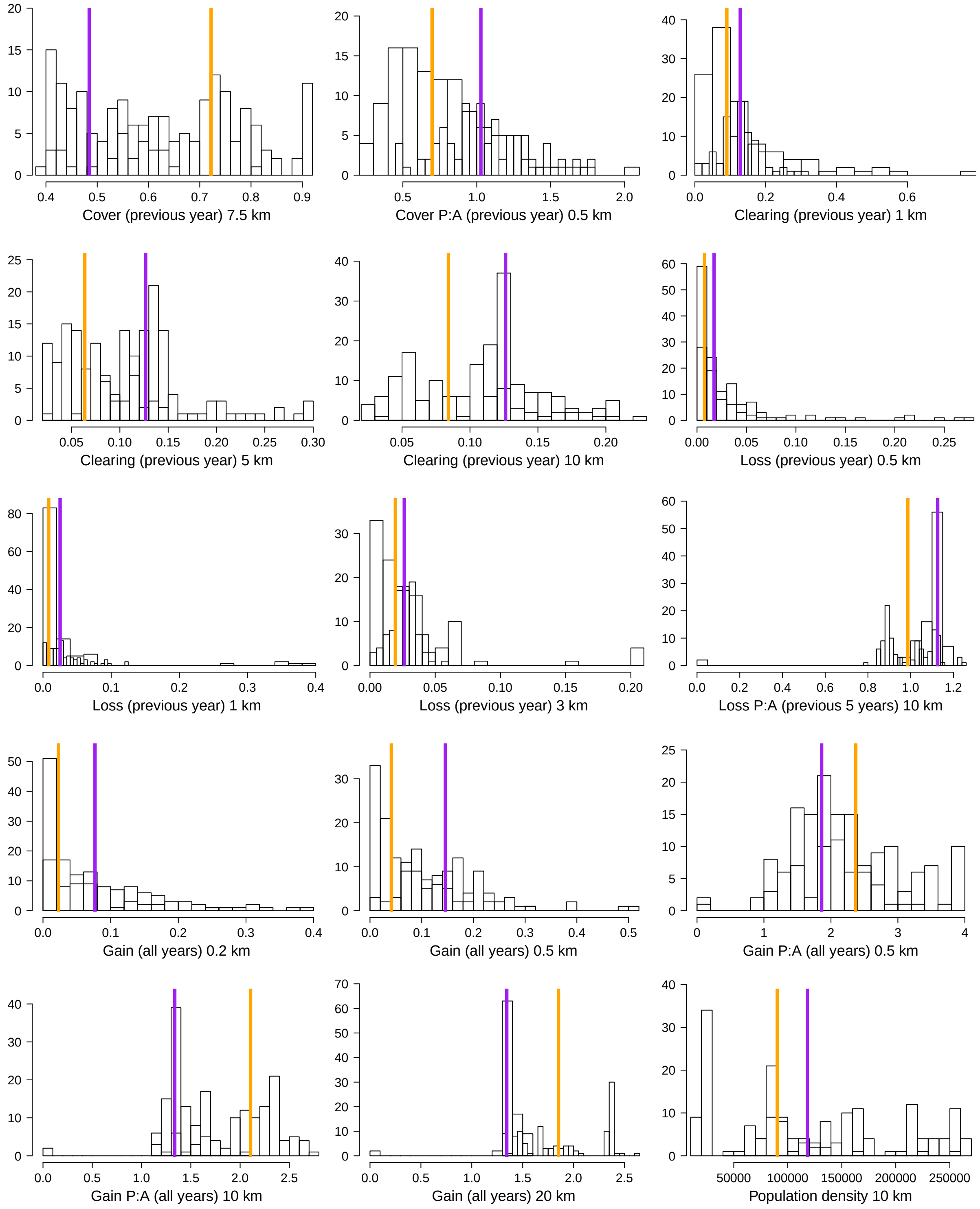

Figure S6. The predictive ability of BRT models of *P. knowlesi* occurrence across spatial scales, with variables summarised over the same single scale (0.1-20 km; 16 predictors per model) and simultaneously over all 11 scales (All; 176 predictors per model). Green and yellow points represent models fitted across the whole study site, blue and purple points models fitted to mainland data only. AUC denotes area under the receiver operator curve.

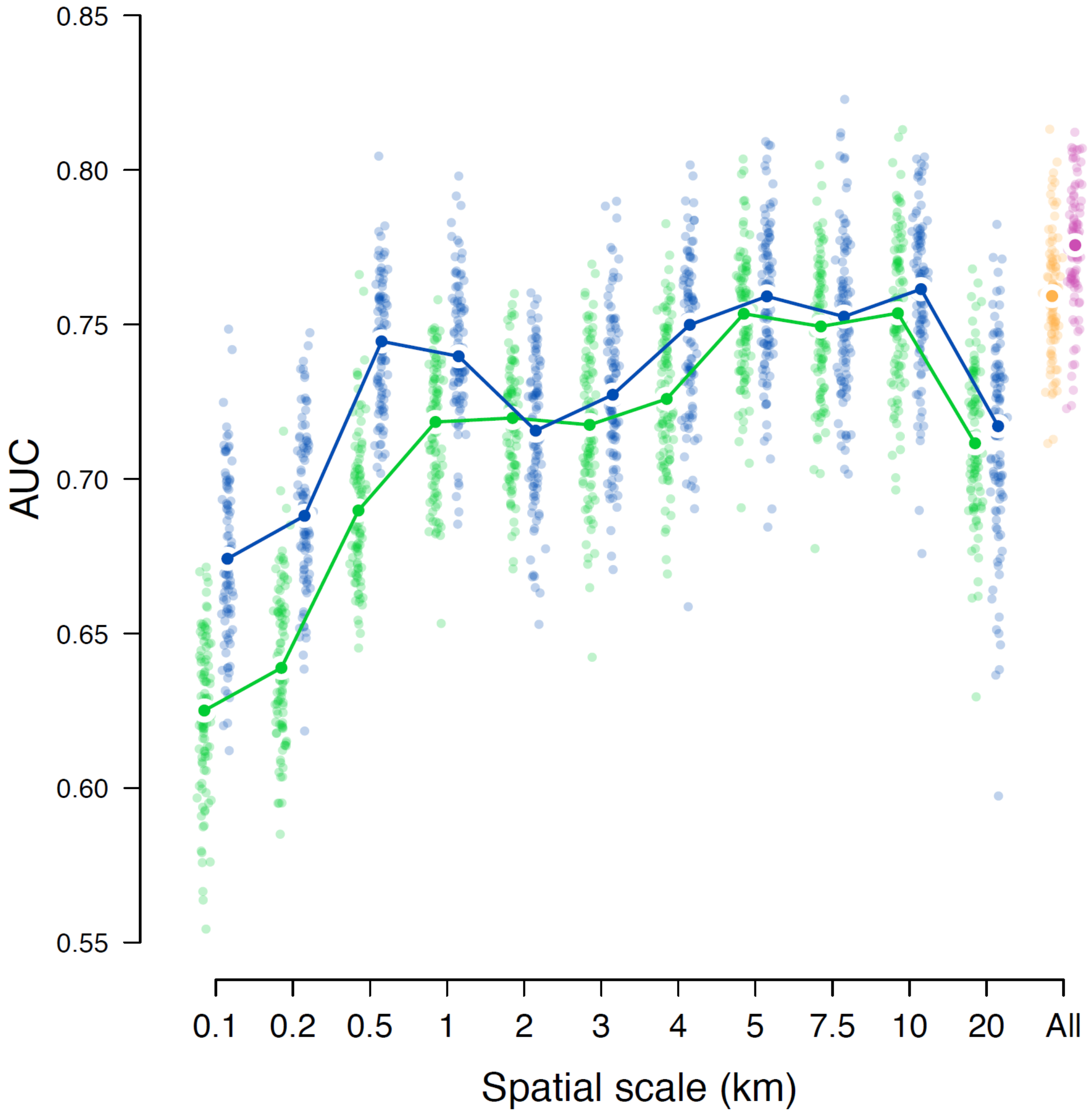

Table S1. Spearman rank correlations comparing relative variance with median relative variable importance (RVI) across spatial scales from the full BRT model fitted across the whole study site (176 predictors).

Variable	Spearman’s rank correlation coefficient	Spearman’s rank correlation test p-value
Cover (previous year)	0.02	0.539
Cover P:A (previous year)	0.91	0.061
Cleared (previous year)	0.05	0.248
Cleared P:A (previous year)	0.76	0.094
Loss (previous 5 years)	-0.49	0.286
Loss P:A (previous 5 years)	-0.44	0.087
Loss (previous year)	-0.17	0.86
Loss P:A (previous year)	-0.22	0.734
Gain (all years)	0.26	0.755
Gain P:A (all years)	0.03	0.734
Mean elevation	-0.31	0.989
Mean aspect	-0.38	0.061
Mean slope	0.36	0.094
Mean NDVI	0.63	0.034
SD NDVI	0.6	0.052
Population density	-0.09	0.924
